# Identification of Long-Distance Mobile mRNAs Responding to Drought Stress in Heterografted Tomato Plants

**DOI:** 10.3390/ijms26073168

**Published:** 2025-03-29

**Authors:** Kanghua Du, Da Zhang, Zhong Dan, Lingfeng Bao, Wanfu Mu, Jie Zhang

**Affiliations:** 1 Tropical Eco-agriculture Research Institute, Yunnan Academy of Agricultural Sciences, Yuanmou 651300, China; kanghuadu@yaas.org.cn (K.D.); zhangda@yaas.org.cn (D.Z.); dz@yaas.org.cn (Z.D.); blf1128@yaas.org.cn (L.B.); sinongmwf@126.com (W.M.); 2 College of Landscape and Horticulture, Yunnan Agricultural University, Kunming 650201, China; 3 Key Laboratory of Vegetable Biology of Yunnan Province, Yunnan Agricultural University, Kunming 650201, China

**Keywords:** graft, tomato, *Solanum pennellii*, mobile mRNAs, drought stress

## Abstract

Grafting is widely used as an effective strategy to enhance tolerance to biotic and abiotic stresses and improve fruit quality in horticultural crops. However, the molecular mechanisms of transcription and the regulatory functions in response to drought stress of mobile mRNAs remain poorly understood. In this study, we developed a grafting system based on the “one grafted plant—three samples” approach using the cultivated tomato/*Solanum pennellii* (Heinz 1706/LA 0716) heterografting system. A bioinformatics pipeline was developed based on RNA-seq to identify mobile mRNAs in the heterografting systems. A total of 61 upwardly and 990 downwardly mobile mRNAs were identified. Furthermore, we found that the mobility of mRNAs was not correlated with their abundance. The functional annotation and enrichment analysis indicated that mobile mRNAs were mainly involved in RNA binding, photosynthesis, photosystem, response to heat, and translation processes, and ultimately increased the drought tolerance of grafted plants. In addition, we also analyzed the RNA-binding proteins (RBPs) of downwardly mobile mRNAs and found that RBPs were conserved among species. Further, mobile mRNAs may be degraded during transportation. This study provides a pipeline for detecting mobile mRNAs in plant heterografting systems and offers new insights into future studies on long-distance mRNAs transport and regulatory mechanisms involved in drought stress responses.

## 1. Introduction

Tomatoes (*Solanum lycopersicum* L.) are one of the world’s major vegetable crops and the second most consumed vegetable globally, holding an important position in the vegetable industry [1,2]. However, tomato growth is influenced by various biotic stresses and diseases as the environmental conditions change. Grafting is a traditional technique in which a part of a plant (the scion) is joined to a part of another plant (the rootstock) to form a new grafted plant [3,4,5,6]. In addition, grafting is an important agricultural measure that effectively increases crop yields, improves quality, and enhances crop resistance for economic benefits, and it also enhances the plant’s ability to defend against both biotic and abiotic stresses [4,7], such as increasing abiotic stresses for salt stress, drought stress, and low-temperature stress [8]. In recent years, a large number of studies have investigated the molecular mechanisms of heterografted tomato plants. The results show that grafting cultivated tomato onto wild tomato enhances the salt tolerance of tomato plants and provide a theoretical basis for sustainable agriculture in saline environments [9,10]. The tomato/goji heterografting system improves tomato quality and flavor by regulating multiple genes involved in the phenylpropanoid, sucrose, and starch pathways in the fruit [11]. With the development of sequencing technology, grafting is not only used to explore the physiological interactions between rootstock and scion but also serves as a tool for screening mobile genes. Therefore, exploring the regulatory mechanisms of mobile genes in plants using grafting is an important and basic biological question.

It is well known that mRNAs play an important role in plants by regulating their growth and development and carrying genetic information [12]. Studies have shown that mRNAs can move long distances via cell-to-cell transport through plasmodesmata (PD) and the phloem vasculature, following the convective flow of phloem streams [13,14,15]. Previous studies have shown that mobile mRNAs (mob-mRNAs) travel from source to sink tissues through the phloem and play important roles in recipient organs [16,17,18,19]. In the *Nicotiana benthamiana*/tomato heterografting system, 183 long-distance mob-mRNAs were identified, and they were involved in the growth regulation of grafted plants [20], while under nitrogen (N), phosphorus (P), and iron (Fe) deficiency conditions, 199, 172, and 211 mob-mRNAs were identified, respectively. The enrichment analysis indicated that long-distance mobile mRNAs were associated with low mineral levels, with some potentially playing crucial roles in hormone metabolism and altered root architecture [21]. Additionally, 138 mob-mRNAs were identified in the *Arabidopsis*/*Nicotiana benthamiana* heterografting system that moved from scion to rootstock [22]. Mobile mRNAs also play a role in regulating abiotic stress in grafted plants. In the cucumber/watermelon heterografting system, 3546 mob-mRNAs were identified as being transported to specific tissues in the phloem tissue under P-deficient conditions to regulate P-elemental homeostasis in plants [19]. Furthermore, in the cucumber/pumpkin heterografting system, 309 mob-mRNAs were identified under drought stress conditions, and these mob-mRNAs might be involved in the regulation of plant photosynthesis and improve the drought tolerance of plants [23]. In the watermelon/bottle gourd heterografting system, a total of 2592 mob-mRNAs were detected under normal and low-temperature stress conditions. It was found that the mobility of mRNAs was independent of their abundance, and that upwardly moving mRNAs may mediate the regulation of abscisic acid, thereby increasing the cold tolerance of plants [24]. 

To date, mRNAs have also been cross-species long-distance translocated between host plants and their hosts. Previous studies have demonstrated the cross-species movement of mRNAs from host plants to parasitic plants, with at least 27 mRNAs shown to be translocated between hosts and *Cuscuta pentagona* [25,26]. The mechanisms of mRNA mobility differ among various host–parasite pairs [27]. Although the long-distance movement of mRNAs between scion and rootstock has been documented in both model and non-model plant heterografting systems, little is known about this phenomenon in the cultivated tomato/wild tomato heterografting system under drought stress. 

In this study, we developed a heterografting system using wild tomato and cultivated tomato to investigate the long-distance movement of mRNAs from scion to rootstock and (root) to rootstock (leaf). Through a systematic biological analysis, a total of 61 upwardly and 990 downwardly mobile mRNAs were identified from source–sink tissues. This study provides new insight into resolving drought tolerance in heterografted plants by analyzing mobile mRNAs expression profiles.

## 2. Results

### 2.1. A Heterograft System for Identifying the Long-Distance Mobile mRNAs

**Figure 1:**
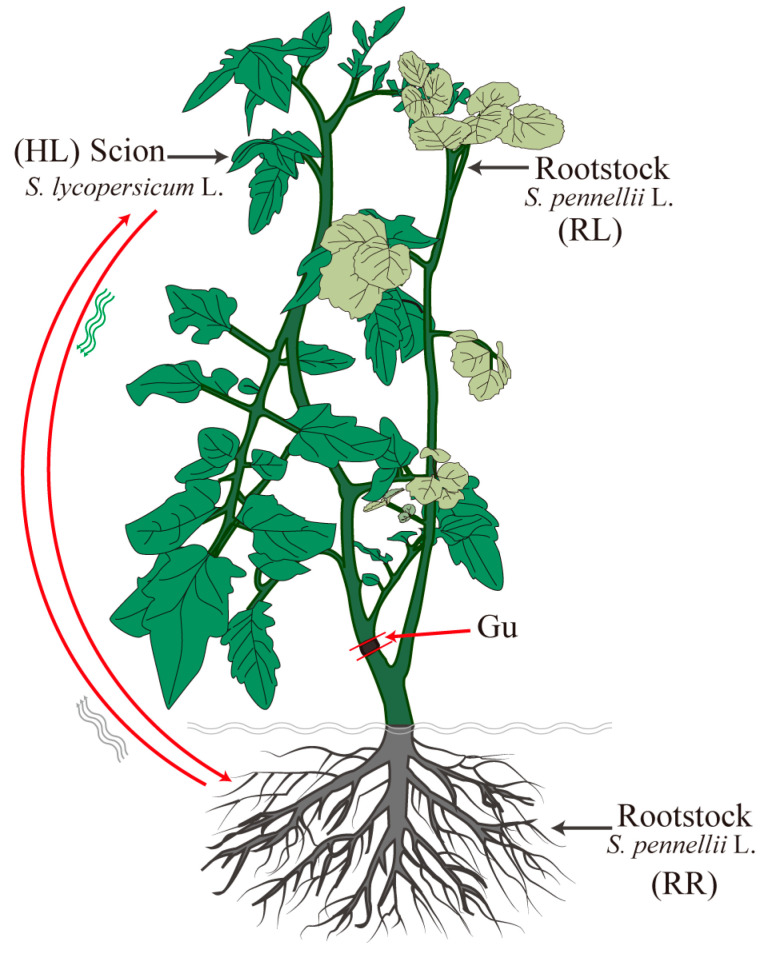
A heterografting system of “one grafted plant—three samples”; cultivated tomato was used as the scion and wild tomato was used as rootstock. HL: The leaf tissue from the scion (Heinz 1706). RR: The root tissue from rootstock (LA 0716). RL: The leaf tissue from the lateral branch of the rootstock.

**Figure 2:**
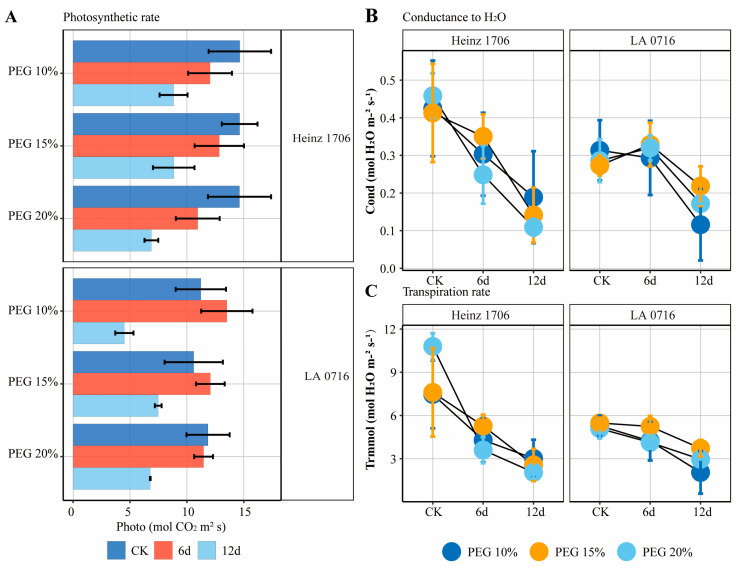
Photosynthesis in grafted plants under drought stress conditions. (**A**) Photosynthesis rate. (**B**) Relative water content in leaves. (**C**) Transpiration rate of leaves.

We established a heterografting system to investigate whether wild tomato as a rootstock could improve the drought tolerance of grafted tomato plants. In this system, wild tomato (*Solanum pennellii* L., LA 0716) was used as the rootstock and cultivated tomato (*Solanum lycopersicum* L., Heinz 1706) was used as the scion (heterografts: Heinz 1706/LA716). The heterograft plants were compared under normal (CK) and drought stresses. Additionally, to explore the respective phenotypic responses of the rootstock and scion to drought stress, we constructed a “scion – rootstock (root) – rootstock (leaf)” graft model (Figure 1). It is worth mentioning that polyethylene glycol 6000 (PEG-6000) was used to simulate plant drought stress and was applied for 12 days. The comparison of control (CK) and drought stress plants showed that the photosynthetic rate, transpiration rate, and relative water content of the leaves decreased with increasing drought severity and duration (Figure 2). Interestingly, it has a similar phenotype of the leaf tissue of the scion (HL) and rootstock (lateral branch of a rootstock, RL). This suggested that the drought signals were first sensed by the root tissue (root tissue of rootstock, RR) and then transmitted to the leaf tissues in grafted plants under the drought stress condition. The leaf tissue reduces the photosynthetic and transpiration rates, thereby reducing plant water loss and enhancing the drought tolerance of the grafted plant.

### 2.2. Long-Distance Movement of mRNAs in Scion to Rootstock

**Figure 3:**
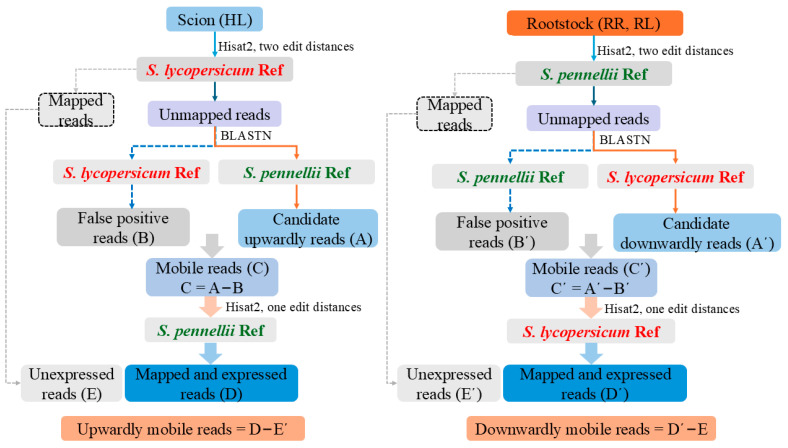
A pipeline of bioinformatic analysis to identify the mobile mRNAs.

**Figure 4:**
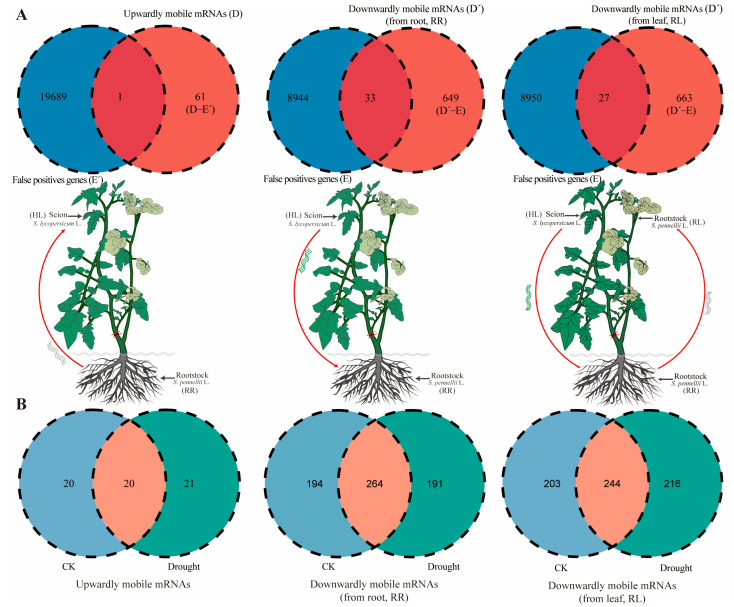
Number of mob-mRNAs in the heterografting system. (**A**) Number of mob-mRNAs from different classes. (**B**) Venn diagram showing upwardly and downwardly mobile mRNAs under CK and drought conditions. The mobile pattern of upwardly mob-mRNAs: rootstock – scion. The mobile pattern of downwardly mob-mRNAs (from root, RR): scion (HL) to GU to rootstock (RR), and downwardly mob-mRNAs (from root, RL): scion (HL) to GU to rootstock (RR) to rootstock (RL).

A pipeline was applied to identify mobile mRNAs (mob-mRNAs) from the RNA-seq data (Figure 3). We obtained 59.2 GB of sequence data from the HL, RR, and RL samples, consisting of approximately 412 million reads. The clean reads were first mapped to the reference genome using HISAT2, resulting in approximately 2,718,718 unmapped reads from scion tissue and 9,929,411 unmapped reads from rootstock tissue. The unmapped reads were then searched against the *S. lycopersicum* Ref and *S. pennellii* Ref using BLASTN, respectively. This yielded an average of 1,505,841 candidate upwardly mobile reads from the rootstock (A) and 5,212,140 candidate downwardly mobile reads from the scion (A’). Additionally, this process yielded an average of 1,451,539 (B) and 4,870,128 (B’) reads as false positives from the homograft reference genome. Subsequently, approximately 24,688 upwardly mobile reads (C) and 609,644 downwardly mobile reads (C’) were mapped to *S. pennellii* Ref and *S. lycopersicum* Ref, respectively. Finally, after filtering out false positive genes, a total of 61 upwardly mobile mRNAs (rootstock to scion) and 990 downwardly mobile mRNAs (649 mob-mRNAs from root tissue of rootstock and 663 mob-mRNAs from leaf tissue of lateral branch of rootstock) were identified (Figure 4A; Table S1).

### 2.3. mRNAs Move from Leaf (Scion) to Root to Leaf (Rootstock)

The movement of mRNAs is specific. Previous studies have shown that mob-mRNAs move from the scion to the rootstock and then return to the scion [20]. In the present study, 663 specific mob-mRNAs were identified in the leaf tissue of the rootstock’s lateral branch (RL). Of these, 341 mob-mRNAs were specific to RL and did not move through root tissues (scion (HL) to GU to rootstock (RL)). Meanwhile, 322 mob-mRNAs moved from the root tissue to the leaf tissue, following the transport model “scion (HL) – rootstock (RR) – rootstock (RL)” (Figure S1). Mob-mRNAs were subjected to degradation during their transport. A total of 327 downwardly mobile mRNAs were detected in the root tissue, but not in the leaf tissue of the rootstock (Figure S1). Under drought stress conditions, a total of 206 downwardly mobile mRNAs were identified from the “scion (HL) – rootstock (RR) – rootstock (RL)” in the RR and RL, with differential abundance observed in the root and leaf tissues of the rootstock (Figure S2; Table S2). Furthermore, some downwardly mobile mRNAs were detectable in RR but not in RL tissues. A possible explanation is that these mob-mRNAs are specific, or the expression abundance is low and undetectable in RL tissues. A similar phenomenon was observed in previous studies [20,28].

### 2.4. Effects of mRNAs Migration Under Drought Stress

**Figure 5:**
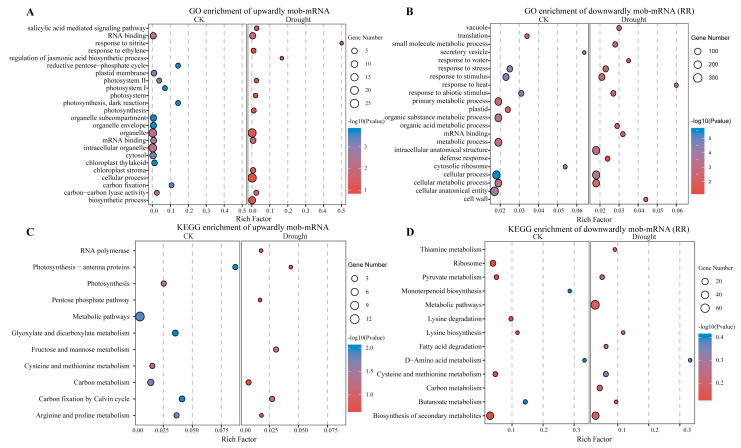
The functional enrichment analysis of mob-mRNAs. (**A**) GO enrichment analysis of upwardly mobile mRNAs, (**B**) and downwardly mobile mRNAs. (**C**) KEGG enrichment pathway of upwardly mobile mRNAs, (**D**) and downwardly mobile mRNAs.

We further analyzed the mob-mRNAs specifically identified under CK and drought stress. The results showed that 21 upwardly mobile mRNAs and 191 downwardly mobile mRNAs in the root tissue of the rootstock were specifically expressed in response to drought conditions, respectively. Likely, 216 specifically downwardly mobile mRNAs were expressed in RL (Figure 4B). The functional enrichment analysis of those mob-mRNAs indicated that the Gene Ontology (GO) terms were predominantly related to RNA binding, photosynthesis, and photosystem and biosynthetic processes from the upwardly mobile mRNAs. In contrast, the downwardly mobile mRNAs were enriched in mRNA binding, response to heat, translation, and defense response (Figure 5A,B; Figure S3A; Table S3). Furthermore, the KEGG enrichment analysis indicated that the mob-mRNAs were mainly involved in the carbon metabolism, photosynthesis, and biosynthesis of secondary metabolites (Figure 5C,D; Figure S3B; Table S4).

### 2.5. Movement of mob-mRNAs Is Independent of Abundance in Source to Sink Tissue

**Figure 6:**
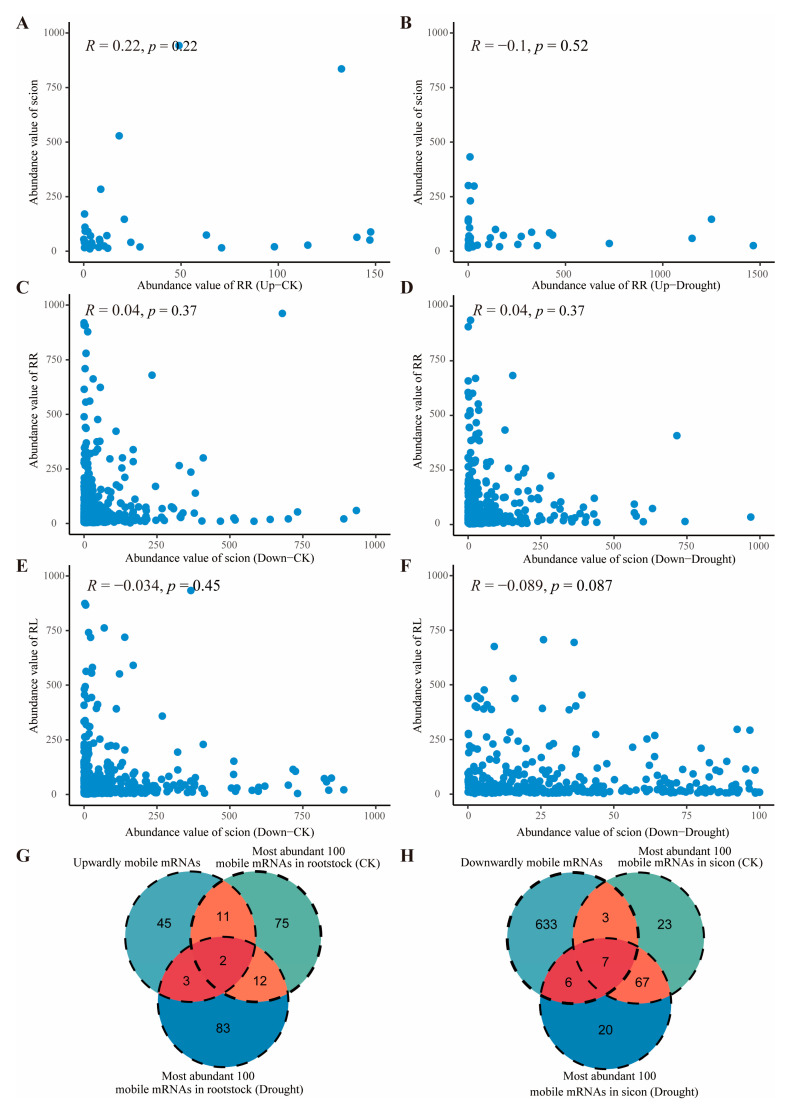
Plot of abundance for upwardly and downwardly mobile mRNAs of the scion and rootstock under CK and drought conditions (**A**–**F**). (**G**) Venn diagram showing the number of upwardly mobile mRNAs among the most abundant 100 mRNAs of the rootstock under CK and drought stress conditions. (**H**) Venn diagram showing the number of downwardly mobile mRNAs among the most abundant 100 mRNAs of the scion under CK and drought stress conditions.

Previous studies have shown that the long-distance mobility of mRNAs is independent of their abundance [20,21,24,29]. However, the issue remains controversial. In our study, we systematically compared the abundance of mob-RNAs in source–sink tissues. The results showed no significant association between the abundance of mRNAs and their mobility, for both upwardly and downwardly mobile mRNAs, under CK and drought stresses conditions (Figure 6A–F).

### 2.6. RNA-Binding Proteins Are Conserved

**Figure 7:**
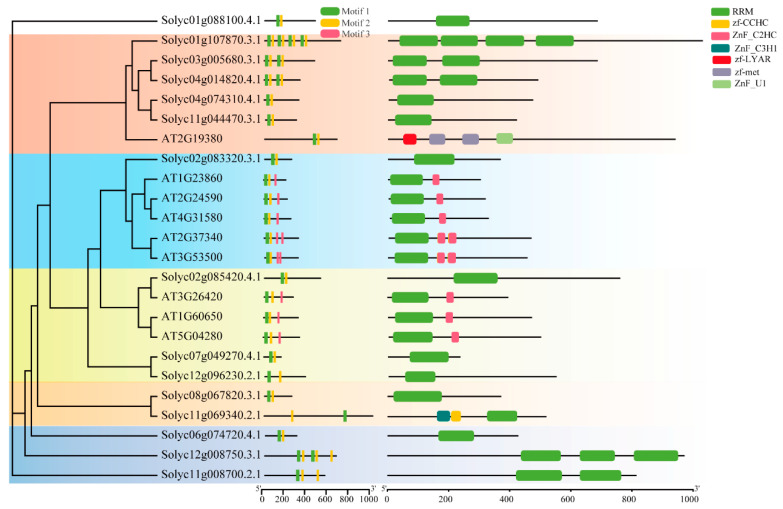
The evolutionary phylogenetic tree and protein structure of RBPs proteins in downwardly mobile mRNAs.

Previous studies have shown that the long-distance transport of RNA is mediated by RNA-binding proteins (RBPs), which are central components of polypyrimidine tract-binding (PTBs) motifs, and these RBPs recognize and bind to mRNAs, triggering the long-distance movement of mRNAs through their own mobility [13,29]. We predicted the RBP structural domains of downwardly and upwardly mobile mRNAs protein sequences by using NCBI-CDD. For the downwardly mobile mRNAs, we identified 15 proteins containing RNA recognition motifs (RRM) or zinc finger structural domains. However, unfortunately, RBPs elements were not found in the upwardly mobile mRNAs sequences. Next, we compared the fifteen RBPs with nine known *AtRBPs* [29]. The phylogenetic analysis showed that RBPs were classified into five subgroups based on their motif and structural domains. The mRNAs that move from scion to rootstock contained only RRM motifs in fourteen of fifteen mob-mRNAs, and only one mob-mRNA (Solyc11g069340.2.1) contained both RRM motifs and zinc finger domains (Figure 7). In addition, a comparison with *AtRBPs* proteins showed that RBPs were also conserved between the two species. Similarly, Fu et. al. [29] demonstrated that RBPs were conserved among eight related species.

## 3. Discussion

Grafting is a technique in which the rootstock and scion are reconnected through the vascular system [30]. In grafted plants, the materials required for the growth and development of the scion are transported through the vascular system, including nutrients and genetic information (mRNAs) [6,18,19]. Furthermore, the phloem tissue contains physiologically active companion cells (CCs) and sieve elements (SEs), which form the CC-SE complex [14]. In plants, the local or long-distance transfer of signaling molecules is mediated by plasmodesmata (PD) and is transmitted between CC and SE through PD [31,32]. Thus, the vascular system and PD together form a transport network for RNA movement across different plant organs [15]. The long-distance transport of macromolecular signals occurs between the rootstock and the scion. Previous studies have focused on the long-distance movement of mRNAs, discovery of mRNAs translocation in phloem tissue by using various technical methods, such as EDTA-facilitated exudation, laser-capture microdissection, fluorescence-activated cell sorting, and MS2-GFP [33,34,35,36,37]. However, the accuracy and authenticity of these mob-mRNAs are still questionable [38]. With the development of genomics technology, a large number of studies have used genomic sequencing to detect large amounts of mobile mRNAs in heterografted plants [8,20,23,29,39].

In this study, we proposed an improved analytical pipeline based on previous studies, which ensured the high reliability of the identified mob-mRNAs (Figure 3). First, the RNA-seq of the rootstock or scion was mapped to the reference genome, respectively. The first mapped set has an edit distance of 2, whereas the second mapped set has an edit distance of 1. Secondly, to ensure the accuracy of the mob-mRNAs, we filtered out false-positive reads, which were identified as originating from homologous sequences using BLASTN. Furthermore, false-positive genes that were not expressed in the source tissues were also excluded. In our study, in addition to using stringent filtering parameter to identify mobile mRNAs (Figure 3), we also constructed a heterografting system using cultivated tomato and wild tomato with large genomic differences (Figure S5). Ultimately, this effectively ensured the high reliability of the identified mobile mRNAs. Previous studies on mob-mRNAs have focused on the movement between scion to rootstock and rootstock to scion [24]. We hypothesize that mRNAs move from the scion to the rootstock and back to the scion; to test this hypothesis, we need develop a new grafting system. For example, potato and *Nicotiana benthamiana* can be grafted onto two separate stems from the same tomato rootstock [20]. However, we used a grafting system to achieve “one grafted plant—three samples” (Figure 1), enabling us to identify three distinct types of downward mob-mRNAs: (1) scion (HL) to rootstock (RR), (2) scion (HL) to rootstock leaf tissue (RL, which did not pass through the root tissue), (3) scion (HL) to rootstock (RR) and rootstock (RL) (Figure S1). This also suggests that the mobile macromolecules, including proteins and transcripts produced from source tissues or CC, were transported into the phloem vascular system through the PD, where they could move to any location in the recipient organs [40]. Previous studies have demonstrated that mob-mRNAs can produce physiological effects in sink tissues and organs, such as leaf, root, and tuber [22,41,42].

Wild tomatoes are used as a rootstock not only to alter plant growth and development but also to enhance the drought tolerance of plants. In addition, a large number of mRNAs were found to move from the scion to the rootstock under drought stress conditions, including specific mobile mRNAs associated with drought stress. A total of 21 drought-up mobile mRNAs and 191 and 216 drought-down mobile mRNAs were found in this study (Figure 4). These mob-mRNAs were involved in RNA binding, photosynthesis, photosystem, response to heat, translation, and photosynthesis pathways in response to drought abiotic stress in grafted plants (Figure 5; Figure S3). Likely, in cucumber/pumpkin grafted plants under drought stress conditions, the mob-mRNAs were involved in carbohydrate metabolism, response to stress, and peroxidase activity [23]. A study showed that the mobile mRNAs *StBEL5* played a growth-promoting regulatory role during potato tuber development [42]. In contrast, the other two sequence-related *StBEL* mRNAs, *StBEL11* and *StBEL29*, functioned antagonistically to repress the target genes of *StBEL5* involved in promoting tuber development [39]. Therefore, we predicted the potential functions of mob-mRNAs in this study. However, the specific functions of mob-mRNAs still requires further validation. 

**Figure 8:**
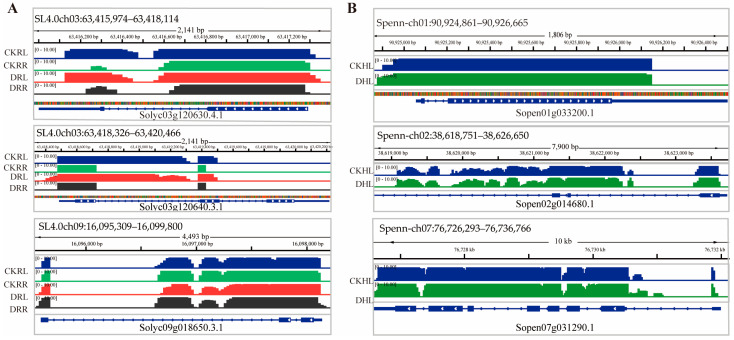
Expression levels and reads coverage of mobile mRNAs in the sink tissue. (**A**) The read coverage of the top 3 downwardly mobile mRNAs. (**B**) The read coverage of the top 3 upwardly mobile mRNAs.

Moreover, the relationship between mRNA mobility and abundance has been a long-standing issue of interest and discussion. Calderwood et al. [43] constructed an abundance model based on long-distance mobile mRNAs in *Arabidopsis* to investigate the potential link between mRNA abundance and mobility. Their study suggested that most mRNAs were transferred, and that this transfer was a result of localized abundance. However, recent studies have shown that mob-mRNAs mobility did not correlate with abundance [20,24,44]. Our study also showed the same results (Figure 6). Furthermore, we classified the expression of mob-mRNAs in the source tissues into six classes: “>1”, “1–10”, “10–100”, “500–1000”, and “>1000” (Figure S4). These results demonstrated that high abundance did not necessarily correlate with high mobility, and that low-abundance mRNAs could also be transported over long distances (Figure 6G,H). Furthermore, we compared the read coverage of the top three downwardly mobile and upwardly mobile mRNAs under different conditions (Figure 8), respectively. We found that two downwardly mobile mRNAs (Solyc03g120630.4.1 and Solyc03g120640.3.1) on chromosome 2 exhibited significant changes under drought stress. This result strongly validates the reliability of the RNA-seq data and the mobile mRNAs identification pipeline.

In addition to cell specificity, the mobility and transport of mob-RNAs may depend on sequence structure and half-life [20,43]. Previous studies have suggested that long-distance mRNAs transport might be mediated by RBPs [13,19], which have several conserved motifs and structural domains, such as RRMs [45] and zinc finger domains [46]. In this study, we performed the structural domain prediction of mob-RNAs using the NCBI-CDD tool, and fifteen downwardly mob-mRNAs were predicted to contain RRM motifs, one of which also contained zinc finger domains (Figure 7). However, none of the upwardly mobile mRNAs were predicted to have RRM. Therefore, we hypothesize that the presence of RBP structural domains may be only one of the factors influencing mRNA movement, which is a complex and multifaceted process involving various molecular mechanisms and regulatory factors.

Additionally, we found that some downwardly mobile mRNAs were detectable in root tissues but not detected in leaf tissues of the rootstock, suggesting that they had a limited half-life and might be degraded during the movement. Similarly, in the study by Xia et al. [20], where *Nicotiana benthamiana* scions were grafted onto the tops of a 2.5 m-tall tomato plant, 854 mobile mRNAs did not move to the root tissue. In an *Arabidopsis* heterografting system, mob-mRNAs were detected in scion stems but not detected in flower tissues of the scion [28]. We also analyzed the expression levels of 202 downwardly mobile mRNAs from the scion to the rootstock (root) and then to the rootstock (leaf) in both root and leaf tissues of the rootstock. The expression levels of these mobile mRNAs were significantly different across different tissues and organs (Figure S2). Furthermore, we propose a hypothesis or future perspective that mobile mRNAs may be spatiotemporally dynamically expressed under different time points, tissues, and growth conditions. For example, specific mobile mRNAs may be expressed in specific tissues or be rapidly degraded after translocation to sink tissues. This has been demonstrated in previous studies [19] on cucumber/watermelon heterografting plants under Pi stress at different time points (short-term: 0–24 h; long-term: ≥7 days) and in different tissues (lamina, major veins, petiole vascular bundles, and internode vascular bundles). The results revealed that mobile mRNAs exhibited highly tissue-specific expression patterns in sink tissues, and Pi stress significantly increased the number of mobile mRNAs. Although early studies have focused on the spatiotemporal dynamic expression of mobile mRNAs, our understanding of their regulatory mechanisms remains limited. Further investigations are required to elucidate the migration patterns, degradation mechanisms, and functional roles of mobile mRNAs.

## 4. Materials and Methods

### 4.1. Plant Grafting and Drought Stress Treatment

A heterografting system was established using cultivated tomato *Solanum lycopersicum* L., Heinz 1706) as the scion grafted onto wild tomato (*Solanum pennellii* L., LA0716). The grafted seedlings were placed in a dark light environment with a relative humidity of 75%. After 15 days of recovery and growth, the grafted seedlings were colonized into the greenhouse. After 20 days, drought stress conditions were simulated using a polyethylene glycol 6000 (PEG-6000, BioSharp, Hefei, China) solution. The test includes control (CK) and drought stress groups, with the drought stress conditions consisting of 10%, 15%, and 20% of the PEG-6000 solution. Similarly, the CK group received the same amount of water. All experimental groups were treated continuously for 12 days. The experiment was conducted at the Tropical Eco-agriculture Research Institute of Yunnan Academy of Agricultural Sciences, Yuanmou, Yunnan, China.

### 4.2. Photosynthetic Rate Determination

The leaf gas exchange capacity and photosynthetic rate of grafted plants were determined by using the LI-6400 (Li-Cor, Lincoln, NE, USA). Measurements were taken between 9 and 11 a.m. under different drought stress conditions for CK: 10% PEG, 15% PEG, and 20% PEG respectively, with treatments lasting for 6 and 12 days.

### 4.3. Sample Collection

After 12 days of treatment, leaf tissues from both the scion and rootstock, as well as root tissues from the rootstock, were sampled from the grafted plants. Specifically, a method of “one grafted plant—three samples” was used in this study (Figure 1), which included leaves tissue from the scion (HL), root tissue from rootstock (RR) and leaf tissue from the lateral branch of the rootstock (RL). A total of six samples were collected, including the control group (CK), CKHL, CKRR, and CKRL, and the drought stress group (20%), DHL, DRR, and DRL, with three biological and three technical replicates.

### 4.4. Total RNA Extraction, RNA-Seq Library Construction and Analysis

In this study, total RNA was extracted using the RNAprep Pure Plant Kit (Tiangen, Beijing, China), and RNA concentration and purity were measured using NanoDrop 2000 (Thermo Fisher Scientific, Wilmington, DE, USA). RNA integrity was assessed using the RNA Nano 6000 Assay Kit of the Agilent Bioanalyzer 2100 system (Agilent Technologies, CA, USA). Sequencing libraries, generated using the Hieff NGS Ultima Dual-mode mRNA Library Prep Kit for Illumina (Yeasen Biotechnology (Shanghai) Co., Ltd., Shanghai, China), were sequenced on an Illumina NovaSeq platform to generate 150 bp paired-end reads. Next, the clean data (clean reads) were obtained by removing reads containing adapters, poly-N sequences, and low-quality reads from raw data using Fastp software (phred cutoff > 20). The paired-end clean reads were mapped to the reference genomes of cultivated tomato (Heinz 1706, SL4.0, https://solgenomics.net/ftp/tomato_genome/annotation/ITAG4.0_release/, accessed on 1 September 2024) [47] and wild tomato (LA0716, v2.0, https://solgenomics.net/ftp/genomes/Solanum_pennellii/, accessed on 1 September 2024) [48] using HISAT2 [49]. Gene count values were calculated by using the featureCounts program [50], and the TPM (Transcripts Per Kilobase of exon model per Million mapped reads) was calculated based on the gene count value.

### 4.5. Identification of Mobile mRNAs

A pipeline of the bioinformatics for the mobile mRNAs identification is shown in Figure 3. Based on previously reported methods [19,20,23,24], we integrated and further optimized these approaches according to the data from in the present study (Figure 3), successfully identifying reliable mobile mRNAs. The paired-end RNA-seq reads from the scion leaf tissue (HL) and rootstock tissue (including root tissue (RR) and rootstock lateral branch leaf tissue(RL)) were first mapped to the *S. lycopersicum* Ref and *S. pennellii* Ref, respectively, by using HISAT2, allowing up to a two-edit distance. The mapped reads were excluded, but they might represent erroneous false-positive genes that are unexpressed (data sets E and E’). The unmapped reads were considered as the potential candidate mobile transcripts by grafting. In addition, to exclude false-positive reads, the unmapped reads from each sample were further searched against the reference genome using BLASTN (E-value 1e-5). For the upwardly mobile mRNAs, if unmapped reads matched *S. pennellii* Ref, these reads would be categorized as candidate upwardly mobile reads (A). Conversely, if the unmapped reads matched the *S. lycopersicum* Ref, these reads would be considered false-positive reads (B). Therefore, only the reads that did not match the *S. lycopersicum* Ref were regarded as upwardly mobile reads. Then, the remaining reads were further mapped to the *S. pennellii* Ref using HISAT2, allowing up to a one-edit distance. Only the genes that matched the *S. pennellii* Ref (D) and excluded false-positive genes (E’) were considered the upwardly mobile mRNAs (D–E’). Similarly, downwardly mobile mRNAs were identified by excluding false-positive reads and unexpressed genes from scion leaf tissue (D’–E). The mob-mRNAs were identified if the corresponding reads were detected in at least two out of the three biological replicates.

### 4.6. Functional Enrichment Analysis of Mobile mRNAs

To predict the potential functions of mobile mRNAs, we performed Gene Ontology (GO) and Kyoto Encyclopedia of Genes and Genomes (KEGG) enrichment analyses. First, the sequences of mobile mRNAs were compared with the protein sequence database of *Arabidopsis* (TAIR10, https://www.arabidopsis.org) using BLAST (E-value set to 1e-5) to identify highly homologous genes, followed by GO and KEGG enrichment analyses.

### 4.7. Analysis of RBPs Structural Domain

The structural domains of long-distance mobile protein sequences were searched in the NCBI Conserved Domains Database (CDD) (https://www.ncbi.nlm.nih.gov/Structure/cdd/wrpsb.cgi) [51]. Protein sequences containing RRM motifs and zinc finger domains were then screened to identify RNA-binding proteins (RBPs) associated with mobile mRNAs. These sequences were compared with nine RNA-binding proteins (RBPs) from A. thaliana [29]. The default parameters were used in the analysis of RBPs.

## 5. Conclusions

In this study, we detected 61 upwardly and 990 downwardly mobile mRNAs in the grafted tomato system. The results indicated that mob-mRNAs were involved in RNA binding, photosynthesis, photosystem, response to heat, translation, photosynthesis, and carbon metabolism pathways by the GO and KEGG enrichment analysis. In addition, the analysis of mob-mRNAs expression levels in source and sink tissues showed that the mobility of mob-mRNAs was independent of their abundance. Furthermore, mob-mRNAs may undergo degradation during their transport from source to sink tissues. Our study provides a method for identifying mob-mRNAs in heterografting systems for horticultural corps and offers valuable insights into the responses of mob-mRNAs to drought stress in heterografted plants.

## Data Availability

The RNA-seq data used in this study have been deposited in the NCBI public database (SRA accession: PRJNA1221965). The pipeline of mobile mRNAs is publicly available in Github (https://github.com/kanghuadu/Mob-mRNAs-workflows, accessed on 18 February 2025).

## Supplementary Materials

The following supporting information can be downloaded at: https://www.mdpi.com/article/10.3390/ijms26073168/s1.
