# Supplementary material for: Identification of Long-Distance Mobile mRNAs Responding to Drought Stress in Heterografted Tomato Plants
Source: Int J Mol Sci. 2025 Mar 29;26(7):3168. doi: 10.3390/ijms26073168 (PMC11989872; doi:10.3390/ijms26073168)
Supplement: Supplementary file 1 [file ijms-26-03168-s001.zip › Supplementary Figure-Author-proof-20250328.pdf]

Identification of Long-distance Mobile mRNAs Responding to Drought Stress in Heterografted Tomato Plants

Kanghua Du [1,2,†], Da Zhang [1,†], Zhong Dan [1], Lingfeng Bao [1], Wanfu Mu [1] and Jie Zhang [2,3*]

[1] *Tropical Eco-agriculture Research Institute, Yunnan Academy of Agricultural Sciences, Yuanmou, Yunnan, 651300, China.*

[2] *College of Landscape and Horticulture, Yunnan Agricultural University, Kunming, Yunnan, 650201, China.*

[3] *Key Laboratory of Vegetable Biology of Yunnan Province, Yunnan Agricultural University, Kunming, Yunnan, 650201, China.*

[*] Corresponding authors: zhangjie@ynau.edu.cn.

[†] These authors contribute equally to this work.

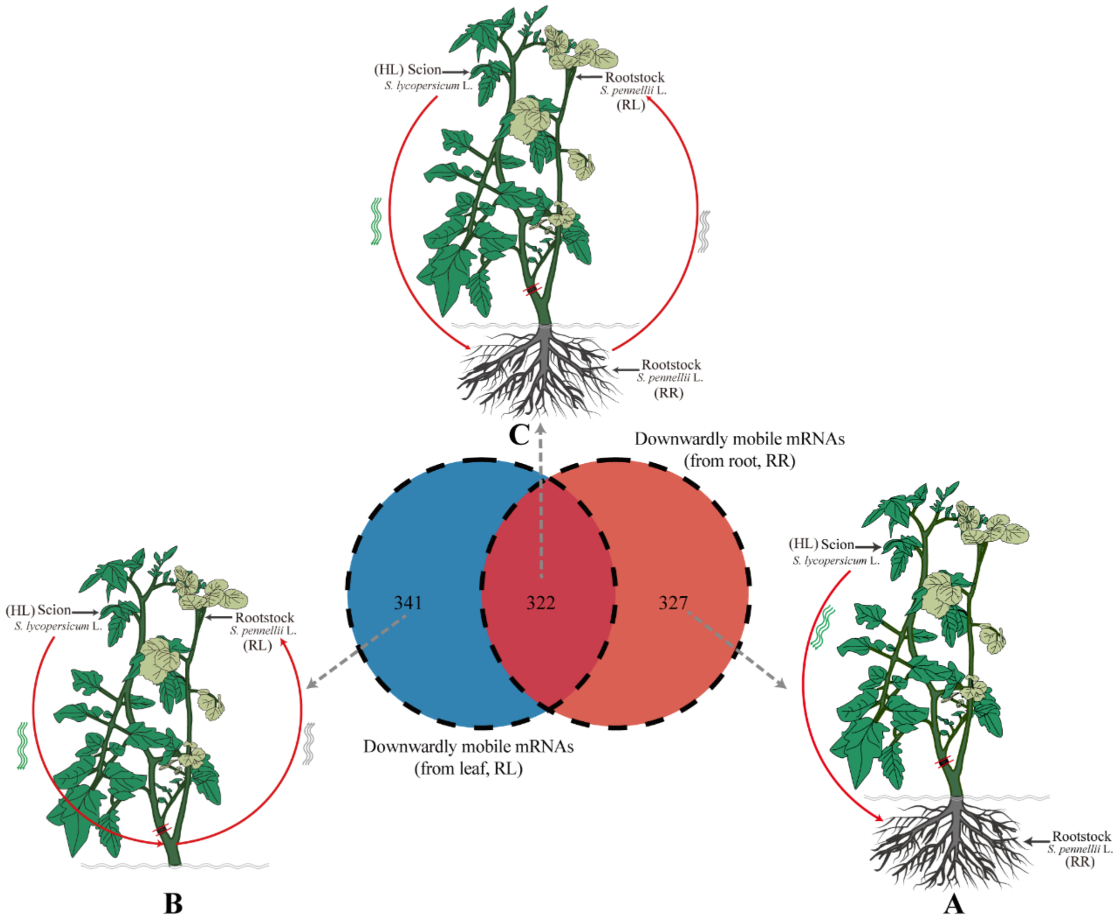

Figure S1. Number and type of downwardly mobile mRNAs. (A) 327 specific downwardly mobile mRNAs from "HL – RR". (B) 341 specific downwardly mobile mRNAs from "HL – Gu – RL". (C) 322 specific downwardly mobile mRNAs from "HL – RR – RL".

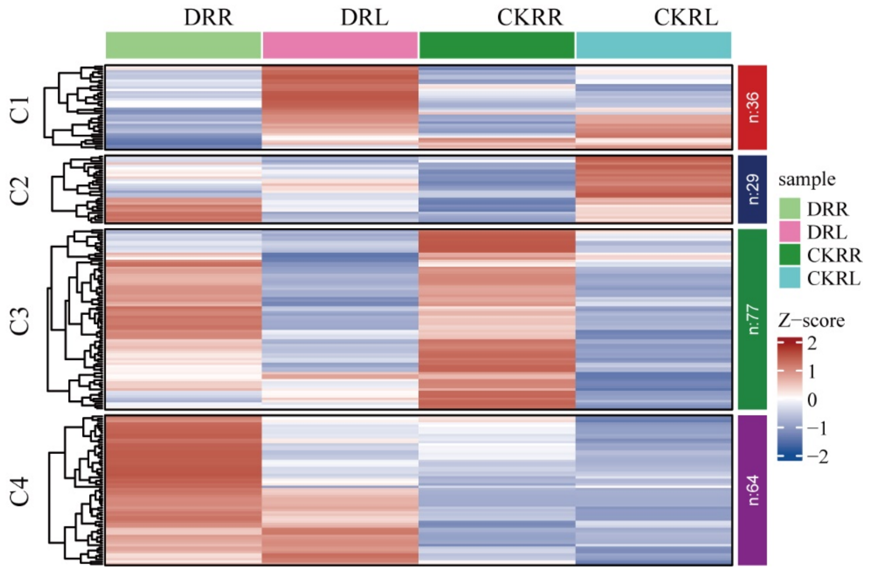

Figure S2. 206 downwardly mobile mRNAs expression levels in root and leaf tissues.

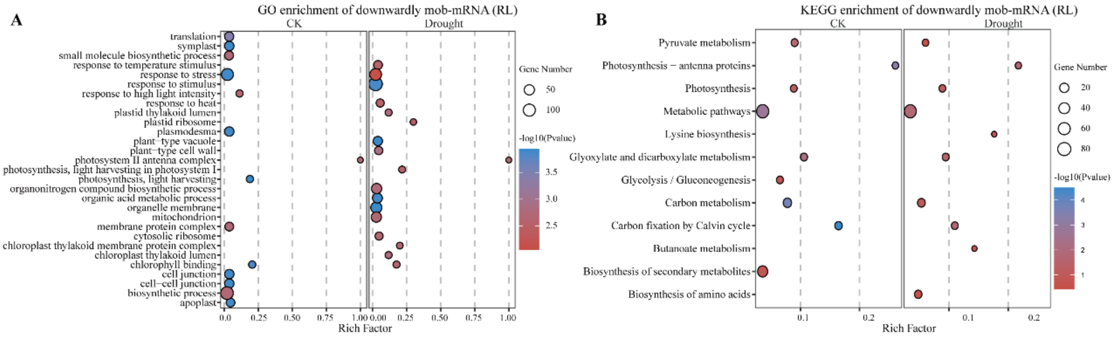

Figure S3. Functional enrichment analysis of downwardly mobile mRNAs from "scion – rootstock (RR) – rootstock (RL)". (A) GO enrichment analysis of downwardly mobile mRNAs. (B) KEGG enrichment pathway of downwardly mobile mRNAs.

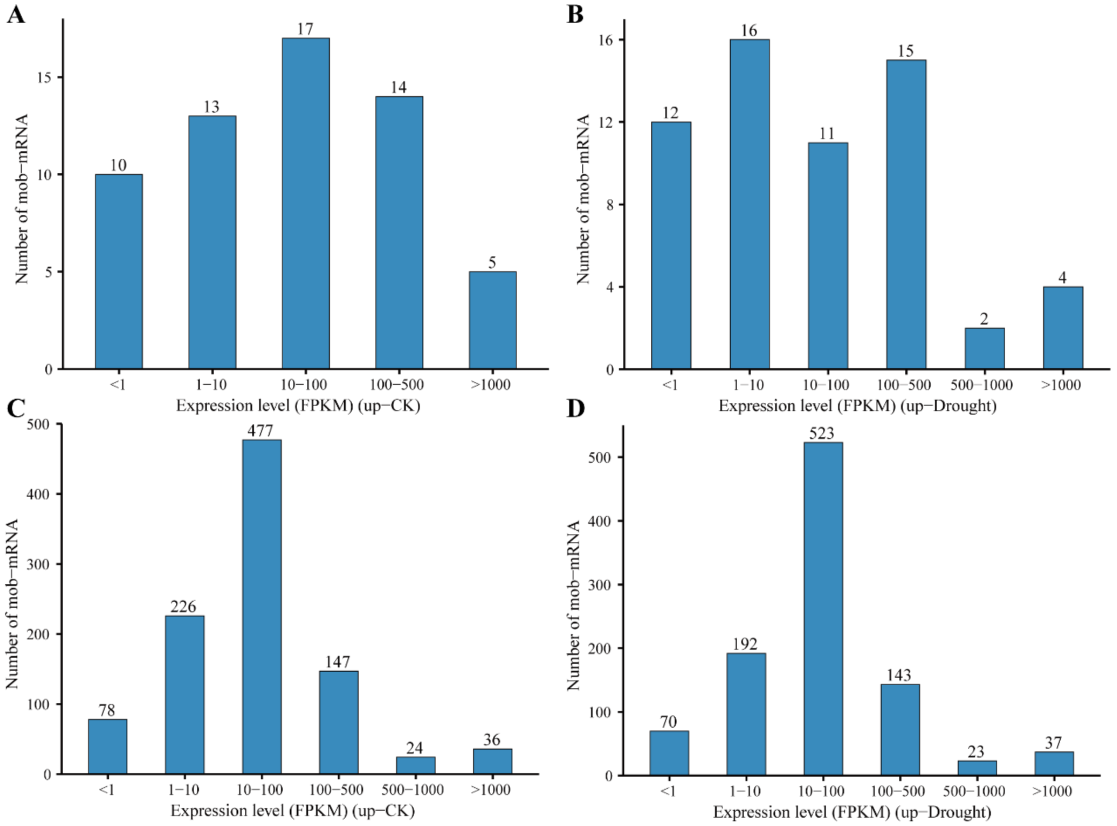

Figure S4. Classification of mob-mRNAs abundance in source tissues. **(A-B)** The classification of upwardly mobile mRNAs abundance in CK and drought conditions. **(C-D)** The classification of downwardly mobile mRNAs abundance under CK and drought conditions.

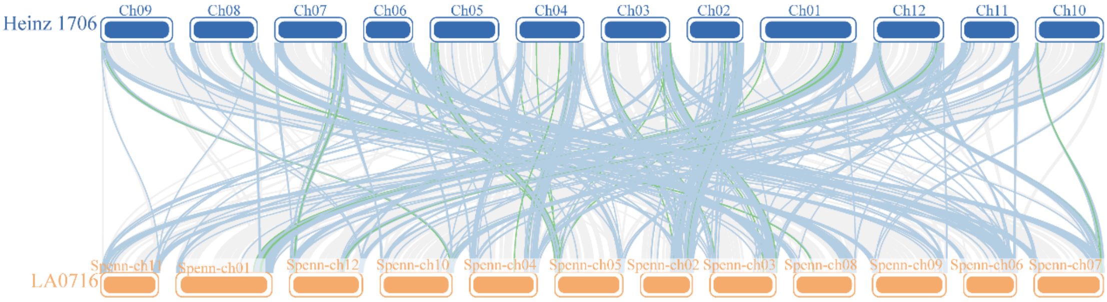

Figure S5. Covariance analysis of the Heinz 1706 genome with the LA0716 genome. Blue lines represent downwardly mobile mRNAs, while green lines represent upwardly mobile mRNAs.
